# Dermatome Mapping Test in the analysis of anatomo-clinical correlations after inguinal hernia repair

**DOI:** 10.1186/s12893-020-00988-1

**Published:** 2020-12-07

**Authors:** Roberto Cirocchi, Isabella Mercurio, Claudio Nazzaro, Angelo De Sol, Carlo Boselli, George Rettagliata, Nicola Vanacore, Alberto Santoro, Domenico Mascagni, Claudio Renzi, Massimo Lancia, Fabio Suadoni, Guido Zanghì, Piergaspare Palumbo, Paolo Bruzzone, Guglielmo Tellan, Piergiorgio Fedeli, Francucci Marsilio, Vito D’Andrea

**Affiliations:** 1 Department of Surgical Science, University of Perugia, Piazza dell’ Università 1, 06100 Perugia, Italy; 2 Inguinal NerveWorking Group, Terni, Italy; 3 General Surgery and Day Surgery, Azienda Ospedaliera Santa Maria Terni, Via Tristano Di Joannuccio, 05100 Terni, Italy; 4 New York Medical College, New York, USA; 5 Istituto Superiore Di Sanità, ISS, Rome, Italy; 6 Department of Surgical Sciences, Sapienza University of Rome, Piazzale Aldo Moro 5, 00185 Rome, Italy; 7 Department of Surgery, Policlinico Vittorio Emanuele University Hospital-General Surgery and Oncology Unit, University of Catania, Catania, Sicily Italy; 8 Dipartimento Di Chirurgia Generale E Specialistica “Paride Stefanini”, Viale del Policlinico, 155, 00186 Rome, Italy; 9 Department of Emergency and Acceptance, Critical Areas and Trauma, “Umberto I” University Hospital, Sapienza University of Rome, 00161 Rome, Italy; 10 Legal Medicine, School of Law, University of Camerino, Camerino, Italy

**Keywords:** Inguinal hernia, Inguinal nerves, Nerve identification, Pain, Follow-up

## Abstract

**Background:**

Nerve identification is recommended in inguinal hernia repair to reduce or avoid postoperative pain. The aim of this prospective observational study was to identify nerve prevalence and find a correlation between neuroanatomy and chronic neuropathic postoperative inguinal pain (CPIP) after 6 months.

**Material:**

A total of 115 patients, who underwent inguinal hernia mesh repair (Lichtenstein tension-free mesh repair) between July 2018 and January 2019, were included in this prospective observational study. The mean age and BMI respectively resulted 64 years and 25.8 with minimal inverse distribution of BMI with respect to age. Most of the hernias were direct (59.1%) and of medium dimension (47.8%). Furthermore, these patients were undergoing Dermatome Mapping Test in preoperatively and postoperatively 6 months evaluation.

**Results:**

Identification rates of the iliohypogastric (IH), ilioinguinal (II) and genitofemoral (GF) nerves were 72.2%, 82.6% and 48.7% respectively. In the analysis of nerve prevalence according to BMI, the IH was statistically significant higher in patients with BMI < 25 than BMI ≥ 25 P (< 0.05). After inguinal hernia mesh repair, 8 patients (6.9%) had chronic postoperative neuropathic inguinal pain after 6 months. The CPIP prevailed at II/GF dermatome. The relation between the identification/neurectomy of the II nerve and chronic postoperative inguinal pain after 6 months was not significant (P = 0.542).

**Conclusion:**

The anatomy of inguinal nerve is very heterogeneous and for this reason an accurate knowledge of these variations is needed during the open mesh repair of inguinal hernias. The new results of our analysis is the statistically significant higher IH nerve prevalence in patients with BMI < 25; probably the identification of inguinal nerve is more complex in obese patients. In the chronic postoperative inguinal pain, the II nerve may have a predominant role in determining postoperative long-term symptoms. Dermatome Mapping Test in an easy and safe method for preoperative and postoperative 6 months evaluation of groin pain. The most important evidence of our analysis is that the prevalence of chronic pain is higher when the nerves were not identified.

## Background

Neuroanatomy of inguinal canal is characterized by great variability [1]. Nerve identification may be difficult because their course or structure may be barely evident [2, 3]. Since inguinal nerves have a relatively superficial course, groin surgery requires particular caution in order to prevent iatrogenic nerve injuries [4, 5]. Failure of local anesthetic procedures and chronic postoperative inguinal pain (CPIP), also known as inguinodynia, are the most common and significant postoperative complications [6]. CPIP is defined as pain persisting beyond the third month after surgical intervention [7] and can affect up to 12% of patients operated on for inguinal hernia. Up to 6% of patients have moderate-severe CPIP which negatively affects their quality of life as it may impact daily activities or, in the worst cases, it may render the patient an invalid [6, 7]. Since inguinal hernias are a quite common and in many countries the Lichtenstein repair is the most frequently used procedure, in particular for primary unilateral groin hernias. CPIP should not be underestimated in planning surgery and patients should always be informed of the eventuality of this post-operative complication [8]. The Lichtenstein tension-free hernioplasty is currently one of the most popular techniques for open repair of inguinal hernias. Local anesthesia is safe and generally preferable for Lichtenstein procedure. Lichtenstein tension-free mesh repair, is based on the following steps:

- Cutaneous incision about 1 cm above and parallel to the inguinal ligament, beginning from the pubic tubercle and extending 5–6 cm laterally.
- Opening of the subcutaneous fat.
- Opening of the Scarpa fascia until the external oblique aponeurosis with accurate visualization of the external inguinal ring and of the lower border of the inguinal ligament.
- Opening of the deep fascia of the thigh to check the femoral canal for a femoral hernia.
- Division of the external oblique aponeurosis from the external ring laterally, preserving the ilioinguinal nerve.
- Mobilization of the superior (protecting the iliohypogastric nerve) and inferior flaps of the external oblique aponeurosis.
- Mobilization of the spermatic cord, along with the cremaster, including the ilioinguinal nerve, the genitofemoral nerve, and the spermatic vessels.
- Opening of the coverings of the spermatic cord and identification and isolation of the hernia sac.
- Inversion (preferred), division, resection, or ligation of the sac, as indicated.
- Placement and fixation of mesh to the edges of the defect or weakness in the posterior wall of the inguinal canal to make a new artificial internal ring.
- Mandatory resection of any nerves that are injured or of doubtful integrity.
- Gentle pulling of the testes back down to their normal scrotal position.
- Closure of spermatic cord layers, the external oblique aponeurosis, subcutaneous tissue, and the skin.
- The operative site is cleaned and a sterile dressing applied. Local infiltration of bupivacaine or ropivacaine may be useful.

The European Hernia Society recommends that in cases where an open approach is indicated (as in case of recurrence after a laparoscopic procedure), the Lichtenstein technique be utilized as the preferred method [6]. Mesh attachment with the use of adhesive glue may be faster and less likely to cause post-op pain if compared to attachment via suture material. Guidelines recommend intraoperative inguinal nerve identification in order to limit or prevent postoperative pain [6] and to reduce the risk of iatrogenic injury, such as entrapment between prosthetic material and tissue [9]. Recently, a systematic review and meta-analysis of worldwide literature, which analyzed identification rates of the three inguinal nerves, found great heterogeneity among studies in particular in Europe [10]. Inguinal nerve identification is performed routinely by few surgeons, despite several studies concluding that this procedure is safe and does not affect operative time [11–14].

Based on these premises, the first aim of this prospective observational study was to identify inguinal nerves during surgery, searching for significant correlations between identification rates during hernia repair and CPIP. The research was undertaken according to the Italian Privacy Laws concerning collection, storage, and analysis of private data. A formal Institutional Research Ethics Board (Perugia University, S. Maria of Misericordia Perugia Hospital and S. Maria Terni Hospital) approval was not required because of the non-interventional, retrospective, and anonymous study design; however, a signed consent for the treatment and the analysis of data for scientific purpose was obtained from all patients or relatives either at admission or as soon as they could give it. The protocol of the study was accepted from Scientific Committee of SICADS (SocietàItaliana di ChirurgiaAmbulatoriale e Day Sugery) and published in the CUDS’ (Club Unità Day Surgery) (web site https://www.clubdaysurgery.it/).

## Methods

### Inclusion and exclusion criteria

Patients included consecutive male patients, aged 18 years and older, undergoing elective open Lichtenstein repair for unilateral primary inguinal hernia. Exclusion criteria: complicated hernias (incarcerated, strangulated, or recurrent); chronic use of analgesics, antidepressants, anxiolytics, anticonvulsants, abuse of alcohol and drugs; fibromyalgia, herpes zoster.

### Outcomes

Primary outcomes: intraoperative identification of the inguinal nerves.

Secondary outcomes: chronic postoperative inguinal pain after 6 months.

### Interventions

All the interventions were performed according to the Lichtenstein technique with a light macroporous mesh in polipropilene. Modification to the original technique consisted in prophylactic inguinal neurectomy (IIN) and mesh fixation with fibrin glue.

### Characteristics of patients

**Fig. 1:**
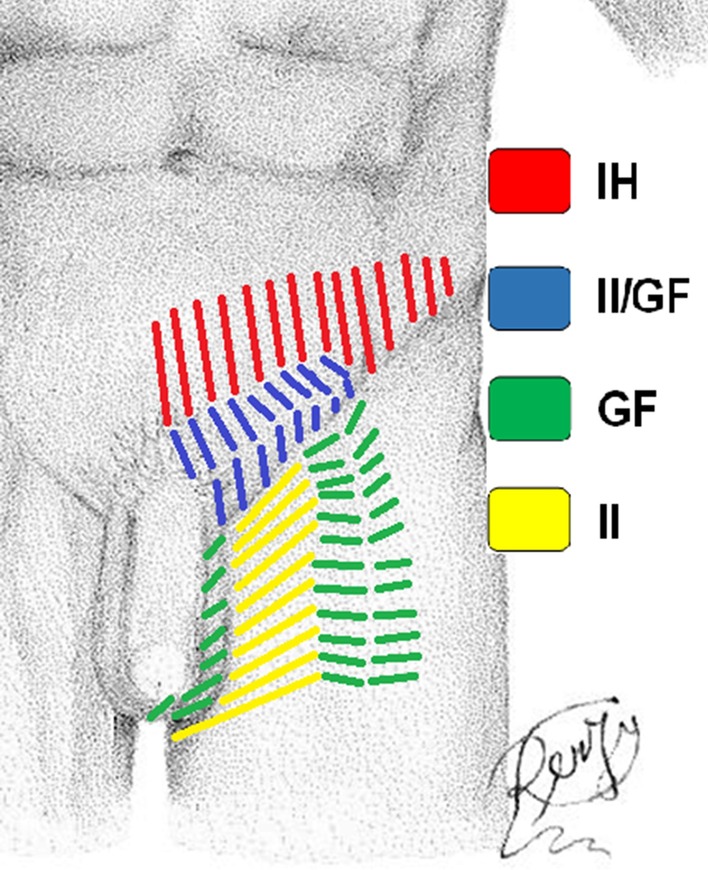
Dermatomes Mapping Test

Before surgery, demographics and anamnesis/comorbidities, which could influence sensitivity for local pain, were collected in an excel table. Furthermore, patients signed an informed consent for the procedure. In addition, they were asked to describe local symptoms and the investigator performed a physical examination. Preoperative pain was localized according to the dermatomal distribution of the three inguinal nerves (Dermatome Pain Mapping Test) (Fig. 1). Pain severity was scored according to the seven criteria of the Inguinal Pain Questionnaire (IPQ) [15]. Intraoperatively, hernia characteristics were recorded according to the EHS (European Hernia Society) classification [6, 16]. Surgeons searched for the iliohypogastric (IH), ilioinguinal (II) and genitofemoral (GF) nerves according to the Lichtenstein procedure. Nerves were isolated; their major diameter was measured in millimeters during the mesh hernia repair. Only the II nerve was cut with scissors and their proximal stumps were tied in order to prevent any hindrance caused by mesh or scar tissues and to reduce the risk of neuroma formation. In our study, the course of the nerves was not recorded.

### Follow-up

**Fig. 2:**
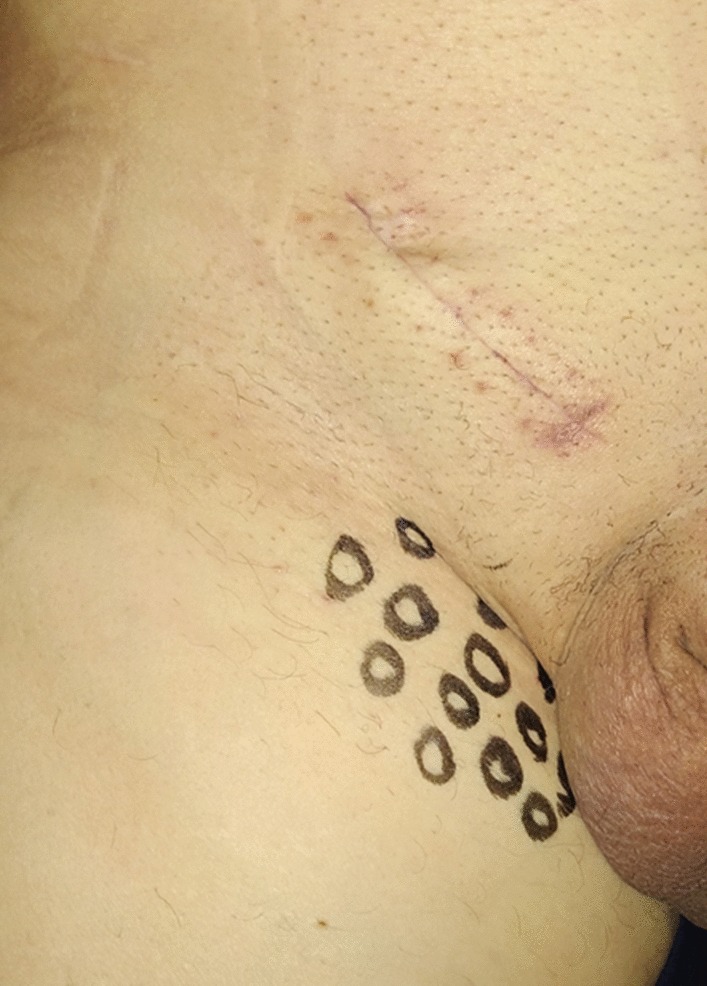
Dermatomes Mapping Test. O = Hypoesthesia or numbness

**Fig. 3:**
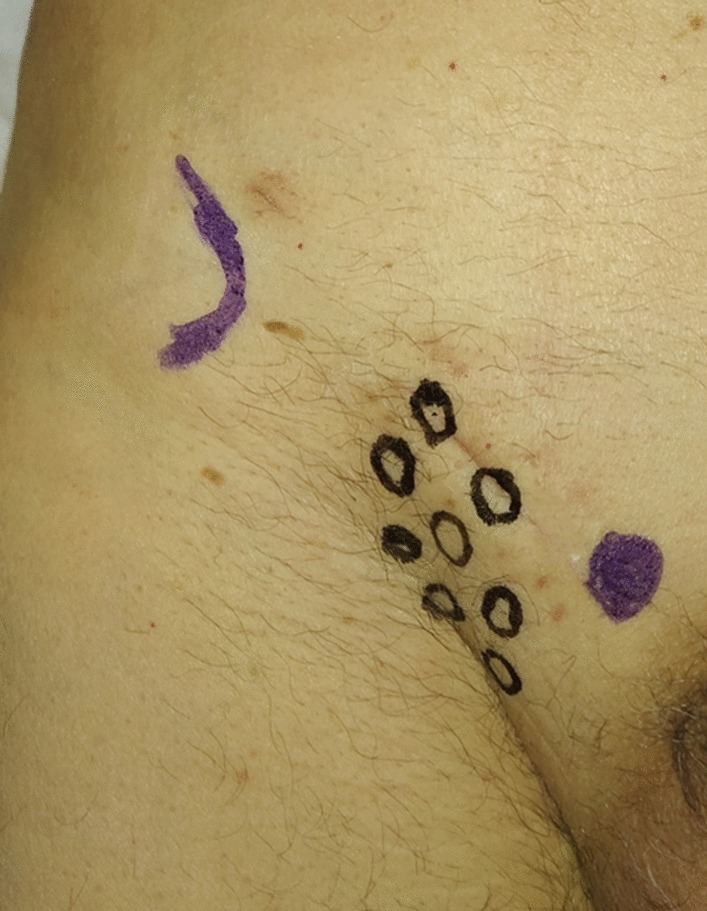
Dermatomes Mapping Test. O = hypoesthesia or numbness

**Fig. 4:**
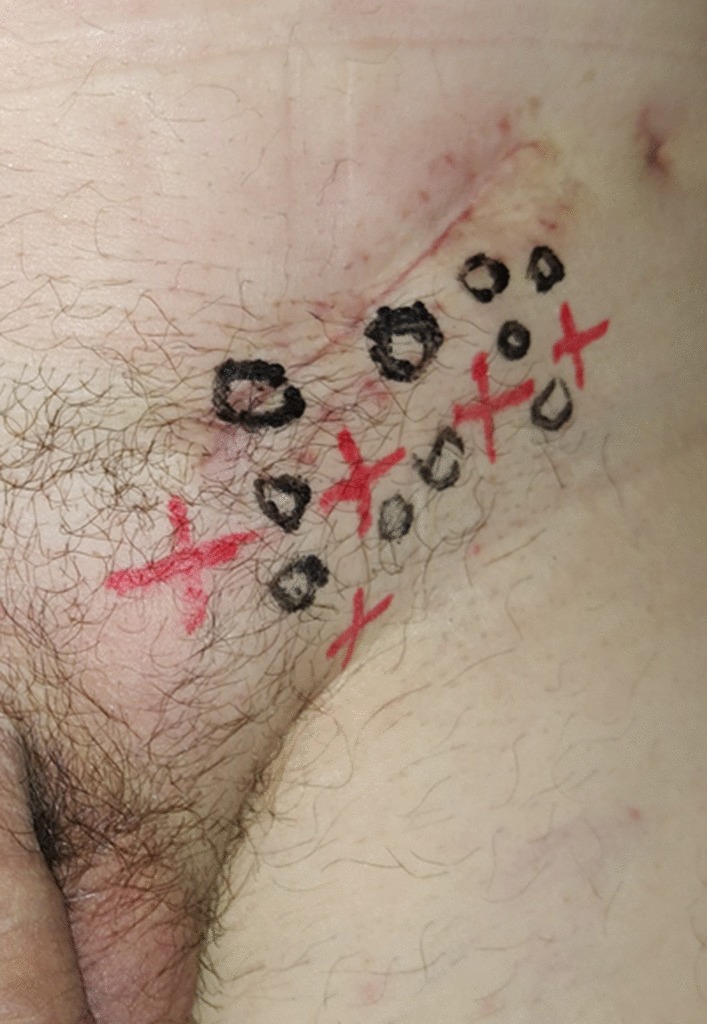
Dermatomes Mapping Test. O = hypoesthesia or numbness. X = pain

**Fig. 5:**
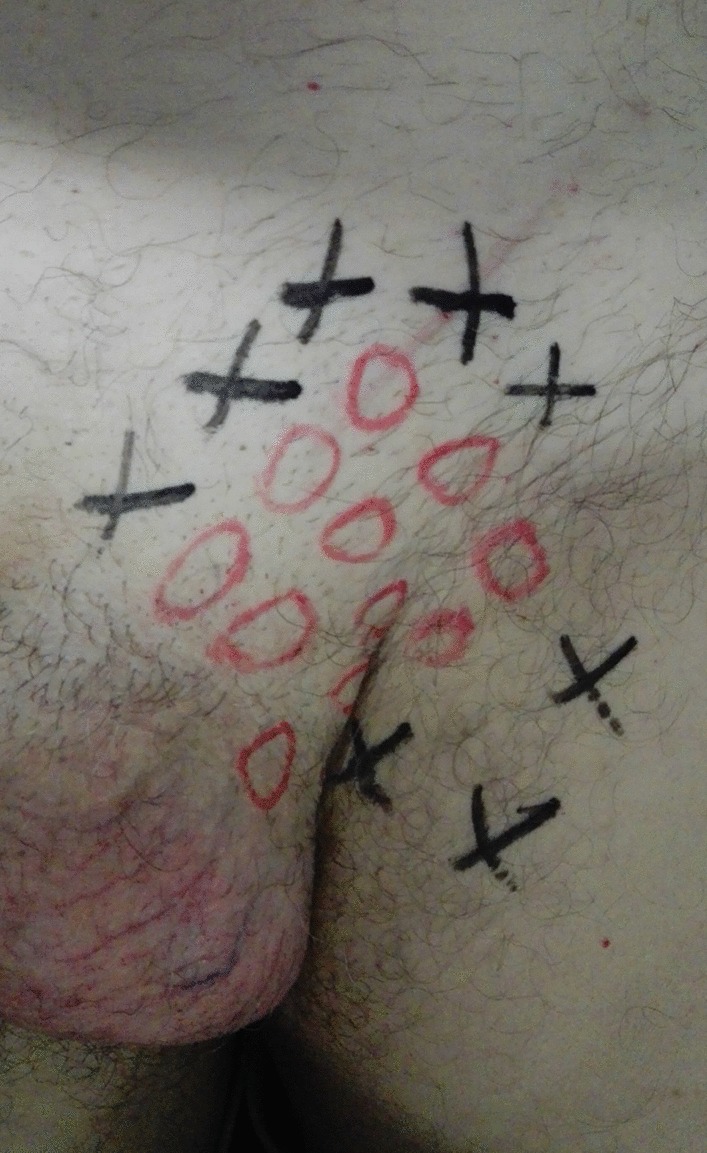
Dermatomes Mapping Test. O = hypoesthesia or numbness. X = pain

Follow-up was set at the sixth postoperative month. Data on local symptoms were collected through a physical examination and by IPQ administration, which was completed in presence of the investigator in order to help with an explanation of the questions [15]. In the presence of pain, regardless of its severity, patients were asked to complete the "Douleur Neuropathique en 4 questionnaire” (DN4) to identify neuropathic pain [17]. A clinical examination was performed preoperatively and postoperatively according to the principles of the dermatome-mapping test (Figs. 2, 3, 4 and 5) [18].

### Statistical analysis

Primary outcomes: Nerve identification rates, separated according to the hernia dimension above-mentioned dichotomous variables, were analyzed with the Chi Square test, setting the confidence interval (CI) to 95% (P < 0.05). The Levene test for the homogeneity of the variance (CI 95%) and Student’s T Test were used to verify the equality of the above-mentioned methods.

Secondary outcomes: Preoperative pain and postoperative (6 months) symptoms were analyzed separately. Sample means of preoperative pain (IPQ scores) were calculated for each nerve separately in the presence (identification) and absence of the nerves, respectively. The Levene test (CI 95%) and Student’s-T Test were used. Correlations between hernia dimension, IPQ score and nerve dimension were tested by Pearson's test.

## Results

A total of 115 patients, who underwent inguinal hernia mesh repair between July 2018 and January 2019, were included in this prospective observational study. The mean age and BMI respectively resulted 64 years and 25.8 with minimal inverse distribution of BMI with respect to age. Most of the hernias were direct (59.1%) and of medium dimension (47.8%) (Table 1).

**Table 1:** Characteristics of patients and hernia

n patients	115				
Age [years; mean (Range, median ± SD^a^)]	64.02 (22–88, 65 ± 15.13) n. pt. ≥ 65: 60 (52.2%)				
BMI [mean (Range, median ± SD^a^)]	25.83 (19.5–41.3, 25.3 ± 3.3) n. pt ≥ 25: 62 (53.9%)				
Radiculopathy [n]	35				
Diabetes [n]	9				
Reumathic disease [n]	4				
Postural issues [n]	40 (heavy workers—7 knee surgery)				
Previous abdominal surgery [n]	54 (29 inguinal hernioplasty)				
Hernia classification (EHS)	*Dimension*	*1*	***2***		*3*
	n. patients	15	**55**		45
	*Type*	*Lateral*		***Medial***	
	n	47 (1:5, 2:20, 3:22)		68 (1:10, 2:35, 3:23)	
	(3 dual, 9 inguinoscrotal)				

Many of the patients were in the age range of between 75 and 84 years (27%), while the 72% of the total were between 55 and 84 years old. Twelve patients (10.4%) were lost to follow-up.

### Identification of the inguinal nerves

The II was the most frequently identified nerve (95 patients, 82.6%). The prevalence of the IH nerve (83 patients, 72%) and GF (56 patients, 48.7%) was lower (Table 2). In the analysis of nerve prevalence according to BMI the IH was statistically significant higher in patients with BMI < 25 than BMI ≥ 25 P (< 0.05).

**Table 2:** Nerve prevalence according to BMI

NERVE	Prevalence [n (% column)]			
	Group A BMI < 25 n.53	Group B BMI ≥ 25 n.62	Series n. 115	P (< 0.05)
IH	44 (83.1)	39 (62.9)	83 (72.2)	0.0164
II	43 (81.1)	52 (83.9)	95 (82.6)	0.70
GF	25 (47.2)	31(50)	56 (48.7)	0.76
IH + II + GF	19 (35.8)	17 (27.4)	36 (31.3)	0.33

No correlation was found between age and nerve detection. Prevalence according to inguinal hernia dimension resulted significantly different for the II nerve in favor of the smallest (≤ 3 cm) (Table 3).

**Table 3:** Nerve prevalence according to inguinal hernia dimension

Inguinal nerve	Prevalence [n (% column)]			
	≤ 3 cm n.70	> 3 cm n.45	Series n. 115	P (< 0.05)
IH	49 (70)	34 (76)	83 (72.2)	0.516
II	62 (89)	33 (76)	95 (82.6)	0.035
GF	35 (50)	21 (47)	56 (48.7)	0.727
IH + II + GF	23 (33)	13 (29)	36 (31.3)	0.654

### Preoperative groin pain

Over half the patients (n.78/115, 67.8%) had preoperative groin pain. Prevalence of pain during activity and rest, which impaired activities, was 65.4% and 35% respectively. This was prevalent in II/GF dermatome (41/78, 52.6%), IH (11/78, 14%) and IH + II/GF dermatomal distribution (11.5%) (Table 4).

**Table 4:** Localization and type of groin preoperative pain

Localization	Type of preoperative pain				
	At rest (%)	During activity (%)	IPQ ≤ 3 (%)	IPQ > 3 (%)	Total
IH	4 (15)	7 (14)	8 (14)	3 (14)	11
II	0 (0)	1 (2)	1 (2)	0 (0)	1
GF	2 (7)	0 (0)	1 (2)	1 (5)	2
II/GF	12 (44)	29 (57)	31 (54)	10 (48)	41
IH + II/GF	4 (15)	5 (10)	6 (11)	3 (14)	9
II + II/GF	2 (7)	3 (6)	4 (7)	1 (5)	5
GF + II/GF	2 (7)	4 (8)	4 (7)	2 (10)	6
IH + II + GF	1 (4)	2 (4)	2 (4)	1 (5)	3
Total (%)	27 (35)	51 (65)	57 (73)	21 (27)	78

Out of three patients with diffuse pain only one had pain influencing activities (IPQ > 3). Pain at rest was prevalent in II/GF dermatome (44%) followed by IH + II/GF localization (15%). Pain during activity resulted more prevalent in II/GF dermatome (57%). Pain with IPQ ≤ 3 prevailed in II/GF region (54%) while IPQ > 3 was less prevalent in this region (48%) in favor of IH + II/GF dermatomes (14%) (Table 5).

**Table 5:** Localization and type of postoperative pain at 6 months

Patient	Preoperative pain		Type hernia EHS	Nerve dimension (mm)			Postoperative pain	
	Localization	Type (IPQ^a^)		IH	II	GF	Localization	Type (IPQ/DN4^b^)
n. 1	IH	Activity 3	L2	2	1	1	IH	2/2
n. 2	II/GF	Activity 2	L2	–	2	1	IH	2/3
n. 3	II/GF	At rest 3	M2	–	2.5	–	IH	2/3
n. 4							Slight hypoesthesia II/GF	
n. 5	II/GF	At rest 4	L3	2	–	–	II/GF	4/4
n. 6							Slight hypoesthesia IH	
n. 7	Heaviness		L1	2	–	1.5	II/GF	2/3
n. 8	Heaviness		L3	2	–	1.5	II/GF	3/4
n. 9	Heaviness		M2	–	3	0.5	II/GF	2/3
n. 10	IH	At rest 4	M1	2	1	1.5	II/GF	3/5
n. 11	GF + II/GF	At rest 3	M2	3	–	–	II/GF	2/4
n. 12	IH + II/GF	Activity 4	M3	2	–	–	II/GF	3/4
n. 13	no pain		M2	3	4	–	II/GF	2/5
n. 14	no pain		M1	–	2	–	II/GF	2/2
n. 15							Slight hypoesthesia II/GF	
n. 16	II/GF	Activity 3	M3	3	3	1.5	II/GF	2/2
n. 17	GF + II/GF	At rest 4	M2	0.5	1	0.5	IH + II/GF	4/5
n. 18	IH + II + GF	At rest 4	M2	4	4	–	II + II/GF	3/4

^a^Inguinal pain questionnaire

^b^Neuropathic pain evaluation DN4 (total score 10, cut-off 4)

### Chronic postoperative inguinal pain after 6 months

**Fig. 6:**
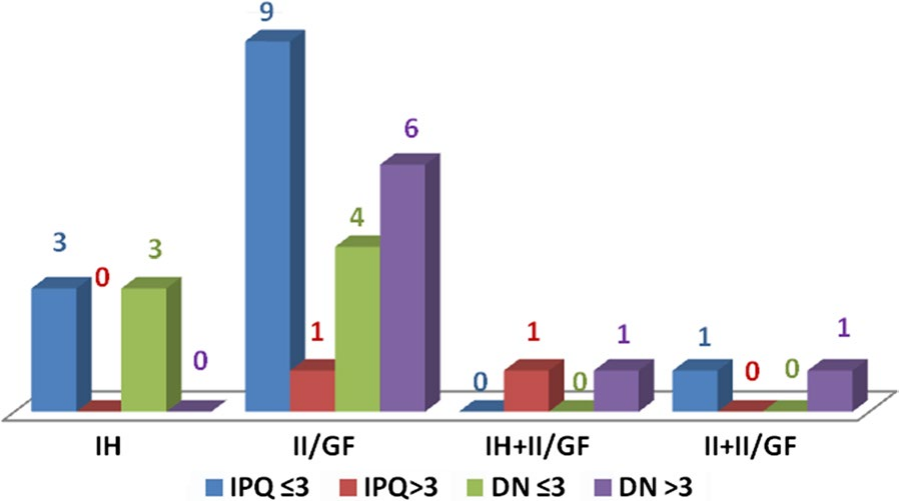
Localization and type of postoperative pain at 6 months

Fifteen patients (13%) had chronic postoperative inguinal pain after 6 months. The CPIP prevailed at II/GF dermatome (66%). In eight patients’ pain was probably of neuropathic origin (DN4 > 3) (53%). Five of these patients presented preoperative pain in the same region and had pain with IPQ > 3. In the other patients pain was probably of nociceptive origin (DN4 ≤ 3) (Table 5, Fig. 6).

### Analysis of 6-month follow-up outcomes with respect to nerve identification and neurectomy

The identification of the IH nerve was performed in 72.2% and the relation between the identification of the IH nerve and chronic postoperative inguinal pain after 6 months was not significant (P = 0.562). When the IH nerve was missing, prevalence of pain was lower (6% vs 12%) and prevalence of hypoesthesia was similar (12–13%). Both the mentioned symptoms prevailed in absence of the IH nerve (6% vs 1%).

The relation between the identification/neurectomy of the II nerve and chronic postoperative inguinal pain after 6 months was not significant (P = 0.542). Narrative analysis is in accordance with the literature since the II resulted the most identified nerve (82.6% in our series vs 84.3% in the literature) and the association of this nerve with pain has been extensively studied. Indeed, prevalence of chronic pain resulted noticeably higher when the II nerve was missing (20% vs 8%): about double the overall incidence of the whole series. Prevalence of the hypoesthesia was slightly higher after neurectomy (13% vs 10%). Both the above-mentioned symptoms were prevalent in the absence of the nerve (5% vs 2%). Most of the patients who resulted negative at follow-up underwent II neurectomy.

### Overall postoperative hypoesthesia

Hypoesthesia was detected in 17 patients (14.8%). No cases of severe hypoesthesia compromising quality of life occurred. This complication was prevalent in II/GF dermatomal distribution (10 pts).

## Discussion

Inguinal nerves have a superficial course and they are exposed to risk of iatrogenic injuries [19]; nerve identification is recommended both to prevent injuries and to perform a more effective local anesthesia [6].

Recently, a systematic review and meta-analysis of worldwide literature reported the identification rates of the inguinal nerves both during open inguinal hernia repair and during cadaveric dissections [10]. Overall prevalence of IH, II, GF nerves resulted 74.2%, 84.3% e 48.2%, respectively. From this review, it is clear that nerve identification during hernia repair is more difficult than in cadaver studies, and greatly depends on the preparedness, expertise and skills of the surgeon [20, 21]. Some studies have tried to find a correlation between BMI (weight) age and cross-sectional area of nerves of the upper and lower limbs [22–24]. In our experience (II 82.6%, IH 72% and GF 48.7%) was in line with the precedent literature. The news of our analysis is the statistically significant higher IH nerve prevalence in patients with BMI < 25. In the literature, it is reported how preoperative pain is related to postoperative pain [25, 26]. Chronic postoperative pain can be influenced by concomitant pain in other regions and by the general physical preoperative state [27]. Inadequate or failure in controlling perioperative acute pain can cause postoperative pain [25, 28]. Furthermore, central sensibilization and neuronal plasticity following intense and prolonged inflammation may predispose to hyperalgesia, thus favoring the passage from acute to chronic pain [8].

In the present study, pain was classified by dermatomal localization through the Dermatome Inguinal Mapping Test and its severity that took into consideration both intensity and impact on activities as well as quality of life with the Inguinal Pain Questionnaire (IPQ) [15]. We aimed to discriminate neuropathic from nociceptive postoperative pain. Nociceptive pain derives from tissue damage or inflammation, which stimulates the nociceptors. It can be subsequent to prosthesis malpositioning, dislocation, “meshoma”, excessive inflammatory response to foreign material or because of periostitis in case of sutures placed over the pubic tubercle. It is usually continuous and present at rest [19]. Neuropathic pain, otherwise, is subsequent to direct or secondary damage of nervous structures. Paresthesia, allodynia, hyperesthesia, hypoesthesia accompanies this type of pain [9]. Neuropathic pain is usually exacerbated by physical activity. Unfortunately, in the literature, a clear differentiation of these two types of pain is difficult to establish since it depends on the diagnostic methodology employed and on the patient’s characteristics [29]. Distension of the peritoneum weakened conjoint tendon and aponeurotic traumas can be possible causes of preoperative pain [30]. Most authors focus on preoperative pain in relation to the II nerve. A recent prospective study supported the hypothesis that preoperative pain can be due to compression neuropathy on the II nerve, with its enlargement in correspondence of the external inguinal ring [31]. The same authors did not find any significant relation between preoperative pain and the type of hernia, or the course of the IH and II nerves [31].

Chronic neuropathic pain is defined as a pain, which lasts beyond the third postoperative month [32]. A systematic review evaluated the prevalence of neuropathic pain in the context of chronic postoperative pain [33]. Neuropathic pain is characterized by a descriptive and a sensorial component. Evaluation of pain considering only these parameters is not adequate since sensitivity alterations may be subsequent to nociceptive stimuli too [33]. For this reason, it is better to consider the neuropathic connotation of pain as probable instead of certain. Probability is higher depending on the strength of the methods employed [34]. Descriptive and sensitivity testing should be completed by specific questionnaires such as the DN4 and a thorough physical examination [17, 33, 34].

The same review reported that the prevalence of chronic (probable) neuropathic postoperative pain was about 31% [33]. In our series, the incidence of probable neuropathic postoperative pain (DN4 = 4) was 33%. This pain was prevalent in II/GF dermatomes. In the majority of patients had CPIP in region of GF nerve, which had the lowest identification rate. The reason for that may be the accidental cut of the nerve intraoperatively. Therefore, the change of strategy into endoscopic procedures in treating many obese patients may be useful to avoid this injury. Indeed, in males the identification of the genital branch of GF nerve is very difficult for its location near cremasteric muscle fibers along the posteromedial spermatic cord and cremasteric vein [35].

## Conclusion

The identification of inguinal nerves is very heterogeneous (II 82.6%, IH 72% and GF 48.7%), for this reason an accurate knowledge of these variations is needed during the open mesh repair of inguinal hernias. The new results of our analysis is the statistically significant higher IH nerve prevalence in patients with BMI < 25; probably the identification of inguinal nerve is more complex in obese patients. In the chronic postoperative inguinal pain, the II nerve may have a predominant role in determining postoperative long-term symptoms, in effect this pain prevailed pain at II/GF dermatome and in 1/3 of cases there is a neuropathic groin pain. Furthermore, Dermatome Mapping Test in an easy and safe method for preoperatively and postoperatively 6 months evaluation of groin pain. The most important evidence of our analysis is that the prevalence of chronic pain is higher when the nerves were not identified.

## Data Availability

The datasets used during the current study are available from the First Author Roberto Cirocchi (mail: roberto.cirocchi@unipg.it) on reasonable request.

## Abbreviations

BMI: Body mass index
CPIP: Chronic postoperative inguinal pain
CUDS: Club Unità Day Surgery
DN4: DouleurNeuropathiqueen 4 questionnaire
EHS: European Hernia Society
GF: Genitofemoral nerve
IH: Iliohypogastric nerve
II: Ilioinguinal nerve
IIN: Ileo-inguinal neurectomy
IPQ: Inguinal Pain Questionnaire
SICADS: Società Italiana di Chirurgia Ambulatoriale e Day Sugery
